# Comparison of sinonasal symptoms in upper respiratory tract infections during the infectious diseases season of November 2023 to March 2024—a cross-sectional study

**DOI:** 10.3389/fmed.2024.1447467

**Published:** 2024-08-29

**Authors:** Marcin Straburzyński, Anna Romaszko-Wojtowicz

**Affiliations:** ^1^Department of Family Medicine and Infectious Diseases, Collegium Medicum, School of Medicine, University of Warmia and Mazury in Olsztyn, Olsztyn, Poland; ^2^Department of Pulmonology, School of Public Health, Collegium Medicum, University of Warmia and Mazury in Olsztyn, Olsztyn, Poland

**Keywords:** sinonasal symptoms, URTI, upper respiratory tract infections, COVID-19, cross-sectional study

## Abstract

**Introduction:**

Upper respiratory tract infections (URTIs) are among the most common reasons for patients consulting a general practitioner (GP) during the infectious diseases season, with viruses being the predominant cause. The COVID-19 pandemic has significantly impacted GPs’ perception of these infections. The pandemic’s progression, especially with the emergence of the Omicron variant, has complicated the diagnosis and treatment of URTIs, with evolving symptoms.

**Aim:**

The aim of this study was to assess the differences in symptoms reported by patients with various infections, such as COVID-19, influenza, common cold, and post-viral rhinosinusitis, during the infectious diseases season of November 2023 to March 2024.

**Materials and methods:**

The study was conducted in a primary health care clinic, providing care for a population of approximately 10,000 people, among adult patients presenting with URTI symptoms during the 2023/2024 infectious diseases season. Patients qualified for the study were swabbed for SARS-CoV-2, influenza A and B and respiratory syncytial virus (RSV) antigens. Symptoms were assessed with the use of a semi-structured questionnaire.

**Results:**

Of the 1810 patients presenting with symptoms of URTIs, 276 patients were included in the study. Among patients with COVID-19, symptoms of nasal obstruction (*p* = 0.005) and nasal discharge (*p* = 0.001) were less common than in those with influenza or common cold. However, these nasal symptoms were significantly more frequent among patients with COVID-19 who had confirmed previous immunization (COVID-19 history or vaccination) (*p* = 0.028).

**Conclusion:**

The incidence of individual sinonasal symptoms varies significantly depending on the aetiological agent of the URTI. This observation may not only help clinicians make the correct diagnosis, but also suggests an inflammatory response in the nasal mucosa and paranasal sinuses that is dependent on the aetiological agent. The study also indicates that this response is altered within the same virus species following immunization.

**Limitations:**

The study’s limitations include a small sample size (276 patients), focus on one season and one GP practice, and reliance on clinical signs and antigen tests. Nonetheless, the findings provide valuable insights. Further research with larger patient groups and extended follow-up periods is required to confirm these findings.

## Introduction

Upper respiratory tract infections (URTIs) are defined as self-limiting irritation and swelling of the upper airways with associated cough and no signs of pneumonia. No other condition that would account for these symptoms is present and the patient has no history of chronic obstructive pulmonary disease (COPD), emphysema, or chronic bronchitis (1). URTIs include conditions such as common cold, laryngitis, pharyngitis/ tonsillitis, acute rhinitis, acute rhinosinusitis and acute otitis media (2). It should be noted that these diagnoses often refer to conditions caused by the same viruses and are used interchangeably, as is the case with common cold and acute viral rhinosinustitis (3). Most URTIs are characterized by similar sinus and nasal symptoms, such as nasal congestion, nasal discharge, and a feeling of facial pressure. Although the clinical presentation is similar, understanding the subtle differences in the clinical picture of these infections can enable physicians to make a quicker and more accurate diagnosis.

The common cold is a viral infection of the upper respiratory tract, defined by the National Institute for Health and Care Excellence (NICE) as “a mild, self-limiting, upper respiratory tract infection characterized by nasal stuffiness and discharge, sneezing, sore throat, and cough.” (4) Acute post-viral rhinosinusitis, as defined by the 2020 European Position Paper on Rhinosinusitis and Nasal Polyps (EPOS), is characterized by symptoms of acute rhinosinusitis (ARS) (i.e., two symptoms, one of which should be nasal congestion and/or discoloured discharge; facial pain/pressure; reduction or loss of smell) that persist for more than 10 days or worsen after 5 days (3).

URTIs have most likely affected humanity for thousands of years, but it was only in the mid-twentieth century that viruses were pinpointed as the aetiological agents of these diseases. Influenza A virus was the first virus to have been isolated in 1933 (5). Since then, extensive research into the aetiology of URTIs, especially during the 1950s and 1960s, has led to the discovery of several other groups of respiratory viruses, such as adenoviruses, rhinoviruses, parainfluenza viruses, respiratory syncytial viruses, enteroviruses, and coronaviruses (6–9). Among all respiratory viruses, rhinoviruses are the most common aetiologic agents in URTIs. It is estimated that rhinoviruses account for approximately 30–50% of all respiratory illnesses annually, but this percentage may rise to 70% during the autumn peak season (10, 11). It is during this period that patients with URTI symptoms constitute the majority of patients in the GP clinics. Although a viral aetiology of URTIs can now be documented in more than 90% of cases owing to modern diagnostic techniques, it is likely that many viruses responsible for respiratory diseases still remain unidentified. The appearance of RT-qPCR assays has made the actual identification of viruses possible. In medical practice, however, for economic reasons, in most cases tests identifying a specific virus strain are not recommended. Hence, most URTIs are diagnosed on the basis of characteristic clinical signs and exclusion of other conditions causing similar symptoms (e.g., allergic rhinitis).

The COVID-19 pandemic has contributed to significant changes in diagnostic and treatment approaches. The widespread availability of RT-qPCR assays and antigen tests has enabled the rapid identification of coronavirus infections, and consequently precise case management and minimising the risk of transmission. The evolution of the coronavirus over the subsequent years, with the Delta variant as the dominant one initially, followed by the Omicron mutation, has brought about changes in clinical signs. The original variant of the SARS-CoV-2 that led to the COVID-19 pandemic was the so-called “wild-type,” characterised by its high infectivity and rapid transmission (12). The Omicron variant first appeared in November 2021. Presently, its newest mutation, the “Kraken” (the Omicron super-variant), is the dominant form of the virus (13, 14).

The aim of this study was to investigate differences in the incidence of sinonasal symptoms between patients with COVID-19, influenza, acute post viral rhinosinusitis and common cold, during the infectious diseases season of November 2023 to March 2024. The main hypothesis was that there are significant differences in the incidence of these symptoms depending on the infection aetiology. In addition, a working hypothesis assumed that prior immunization (history of COVID-19 or vaccination) may alter the incidence of some symptoms.

## Materials and methods

### Study design

This cross-sectional study was conducted at a primary health care clinic, providing care for a population of approximately 10,000 individuals.

### Inclusion criteria

Consecutive patients seeking medical consultation for acute URTI symptoms, including anterior/posterior nasal discharge, nasal congestion, sore throat, fever, and myalgia, within 2 weeks of onset were enrolled in the study. Patients with multiple URTIs were eligible if they reported a symptom-free period of at least 3 weeks between infections.

### Exclusion criteria

Patients were excluded if they presented with isolated general symptoms without signs of upper respiratory tract inflammation, a history of >3 URTI during the previous 6 months, chronic respiratory disorders, immunodeficiency, conditions hindering examination, chronic headache or facial pain, diagnosis of acute bacterial URTI, or lack of symptom resolution after specified durations (4 weeks in URTI, 12 weeks in acute post-viral rhinosinusitis).

### Assessment

First, a general practice nurse assessed patients for URTI symptoms. Those presenting symptoms were further evaluated by a general practitioner (GP) on the same day. Data comprising history and physical examination were collected with the use of a semi-structured questionnaire, focusing on URTI symptoms, accompanying symptoms, and physical examination parameters.

### Diagnosis

Diagnosis of acute viral URTI was confirmed based on inclusion criteria and physical examination findings, along with positive antigen swab test results for COVID-19, influenza A/B virus, or RSV (CorDx Test COMBO: COVID-19 positive predictive value (PPV) 89,09%, negative predictive value (NPV) 100,00%, Influenza A: PPV 100,00%, NPV 99,34%, Influenza B: PPV 96,00%, NPV 99,60%, RSV: PPV 98,98%, NPV 99,21%) (3). Previous COVID-19 immunization status was verified in the national electronic database where positive swab tests results and vaccination information are stored. Acute rhinosinusitis (ARS) was diagnosed according to current European guidelines requiring the presence of at least two symptoms out of: nasal discharge, nasal congestion, hyposmia/anosmia, facial pain/pressure (3). Follow-up telephone consultations were conducted to confirm symptom remission.

### Study duration

The occurrence of URTIs is characterized by marked seasonality, with the highest incidence observed in autumn and winter, especially in temperate regions of the northern hemisphere (15).

Consequently, the study was conducted during one infectious diseases season, spanning from November to March in 2023/2024.

### Statistical analysis

Statistical analysis was performed using R statistical environment version 3.6.0, the PSPP program, and MS Office 2019. A significance level of *p* < 0.05 was adopted. Chi-square tests were used for nominal or ordinal data, Fisher’s exact test for smaller tables, and non-parametric tests for quantitative values. Logistic regression analysis was employed to verify relationships between groups.

## Results

A total of 1810 patients presenting with URTIs symptoms were initially screened. Exclusion criteria were applied, resulting in the exclusion of patients not meeting URTI or acute post-viral rhinosinusitis criteria (*n* = 871), minors (under 18 years of age), those with neurological or psychiatric disorders (*n* = 124), chronic or recurrent upper respiratory tract disorders (*n* = 128), and those diagnosed with acute bacterial URTI (*n* = 7). Ultimately, 276 volunteers were included in the study who were diagnosed with: COVID-19 (*n* = 107/ 38.8%), Influenza A (*n* = 36/13.0%), ‘common cold’ (*n* = 103/37.3%) or APVRS (*n* = 30/ 10.1%). No coinfections were confirmed.

The study, which was carried out in a primary health care clinic, identified differences in the occurrence of URTI symptoms and the relationship between the course of the disease and its different types by analysing data collected from patients with different URTI types.

### Facial pressure

Facial pressure was least frequent in patients with common cold (29.1%, *n* = 30), influenza (27.8%, *n* = 10) and COVID-19 (23.4%, *n* = 25), and was most common in patients with acute post-viral rhinosinusitis (53.3%, *n* = 16). Statistically significant (*p* = 0.017, *χ*^2^ = 10.216, df = 3) higher incidence of facial pressure in patients with acute post-viral rhinosinusitis as compared with other patients was revealed.

### Nasal discharge

Nasal discharge was most often found in patients with common cold (88.3%, *n* = 91), influenza (91.7%, *n* = 33), and acute post-viral rhinosinusitis (96.7%, *n* = 29). It was significantly less frequent (*p* = 0.001, *χ*^2^ = 43.230, df = 3) in patients with COVID-19.

### Nasal congestion

Nasal congestion was most common in patients with common cold (62.1%, *n* = 64), influenza (66.7%, *n* = 24), and acute post-viral rhinosinusitis (70.0%, *n* = 21), and less common in those with COVID-19 (57.0%, *n* = 61), which was statistically confirmed (*p* = 0.005, *χ*^2^ = 12.979, df = 3).

Among patients with common cold, rhinoscopy most often indicated serous inflammation (45.1%, *n* = 46), similarly to those with acute post-viral rhinosinusitis (43.3%, *n* = 13). The findings were most often normal among patients with influenza (50.0%), whereas among those with COVID-19 they were equally normal and indicative of serous inflammation (45.5%, *n* = 15 each). The differences were not statistically significant (*p* = 0.556, *χ*^2^ = 4,906, df = 6). Rhinoscopy findings did not reveal a statistically significant correlation with URTI types.

ARS diagnostic criteria were most often met in patients with common cold (78.6%, *n* = 81), influenza (88.9%, *n* = 32), COVID-19 (64.5%, *n* = 69), and as per definition in acute post-viral rhinosinusitis (100.0%, *n* = 30). However, statistically significant differences (*p* < 0.05) were observed: ARS was least common in patients with COVID-19 (*p* = 0.001, *χ*^2^ = 21.325, df = 3).

**Figure 1:**
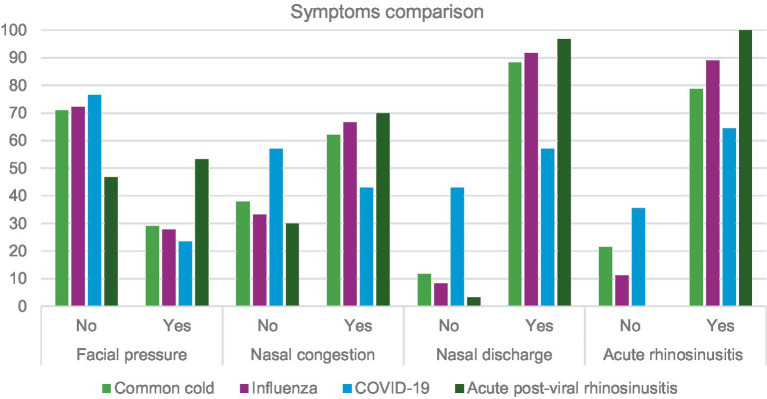
Symptoms comparison based on type of URTI.

Table 1 and Figure 1 presents a summary of data regarding URTI symptoms considering four conditions: common cold, influenza, COVID-19 and acute post-viral rhinosinusitis. A further analysis is presented in Table 2. Where sensitivity and specificity of each sinonasal symptom is presented. This analysis indicates that nasal discharge indicates foremostly COVID-19 while facial pressure is strongly indicative of APVRS.

**Table 1:** Summary of URTI symptoms by condition.

			URTI type				Test findings
			Common cold	Influenza	COVID-19	Acute post-viral rhinosinusitis	
Facial pressure	No	*N*	73	26	82	14	*χ*^2^ = 10,216, *df* = 3, *p* = 0.017
		%	70.9%	72.2%	76.6%	46.7%	
	Yes	*N*	30	10	25	16	
		%	29.1%	27.8%	23.4%	53.3%	
Nasal discharge	No	*N*	12	3	46	1	*χ*^2^ = 43.230, *df* = 3, *p* = 0.001
		%	11.70%	8.30%	43.00%	3.30%	
	Yes	*N*	91	33	61	29	
		%	88.30%	91.70%	57.00%	96.70%	
Nasal congestion	No	*N*	39	12	61	9	*χ*^2^ = 12.979, *df* = 3, *p* = 0.005
		%	37.90%	33.30%	57.00%	30.00%	
	Yes	*N*	64	24	46	21	
		%	62.10%	66.70%	43.00%	70.00%	
		%	88.30%	77.80%	83.20%	80.00%	
	Yes	*N*	12	8	18	6	
		%	11.70%	22.20%	16.80%	20.00%	
Rhinoscopy	Norm	*N*	45	18	15	10	*χ*^2^ = 4.906, *df* = 6, *p* = 0.556
		%	44.10%	50.00%	45.50%	33.30%	
	Serous inflammation	*N*	46	13	15	13	
		%	45.10%	36.10%	45.50%	43.30%	
	Purulent inflammation	*N*	11	5	3	7	
		%	10.80%	13.90%	9.10%	23.30%	
ARS	No	*N*	22	4	38	0	*χ*^2^ = 21.325, *df* = 3, *p* = 0.001
		%	21.40%	11.10%	35.50%	0.00%	
	Yes	*N*	81	32	69	30	
		%	78.60%	88.90%	64.50%	100.00%	

**Table 2:** Diagnostic value of sinonasal symptoms in different URTIs.

		Sensitivity (%)	Specificity (%)	PPV (%)	NPV (%)
Facial pressure	Common cold	29.1	70.5	37.0	62.6
	Influenza	27.8	70.4	12.3	86.7
	COVID-19	23.4	66.9	30.9	57.9
	APVRS	53.3	73.6	56.1	71.3
Nasal discharge	Common cold	88.4	28.9	42.5	80.7
	Influenza	91.7	24.6	15.4	95.2
	COVID-19	57.0	81.6	66.3	75.0
	APVRS	96.7	24.8	44.9	92.2
Nasal congestion	Common cold	62.1	47.4	41.3	67.8
	Influenza	66.7	45.4	15.4	90.1
	COVID-19	43.0	50.0	35.3	58.0
	APVRS	70.0	45.5	44.9	70.5

PPV, positive predictive value; NPV, negative predictive value.

### The influence of prior immunization on the occurrence of ARS in patients with COVID-19

A statistically significant (Table 2) relationship was found between a history of COVID-19 or vaccination against it (prior immunization) and the occurrence of ARS symptoms. Patients with a history of COVID-19 or vaccinated against it more frequently presented with ARS symptoms as compared to those without prior immunization (Table 3).

**Table 3:** Influence of prior COVID-19 immunization on ARS occurrence.

			Prior COVID-19 immunization		Test result
			NO	YES	
Sinonasal symptoms	NO	*N*	34	4	*χ*^2^ = 4.799, *df* = 1, *p* = 0.028
		%	41.5%	15.4%	
	YES	*N*	48	22	
		%	58.5%	84.6%	

## Discussion

One of the significant challenges in clinical practice is differentiating COVID-19 from other URTIs based on symptoms alone. The overlapping symptomatology, particularly with the Omicron and subsequent variants presenting with common cold-like symptoms, makes it difficult to distinguish COVID-19 without laboratory confirmation. Symptoms such as nasal congestion, rhinorrhea, sore throat, and headache are common across various URTIs, complicating the clinical diagnosis. This overlap necessitates a high index of suspicion and often relies on specific diagnostic tests, such as RT-qPCR or rapid antigen tests, to confirm COVID-19. In clinical practice, the symptoms reported by the patient and the pathologies identified during the physical examination are of fundamental importance in the diagnosis, differentiation, and proper treatment of patients. The study, conducted from November 2023 to March 2024, demonstrates differences in the incidence of symptoms such as facial pressure, nasal discharge, palpation tenderness and acute sinusitis according to URTI types. The symptoms of common cold, influenza or acute post-viral rhinosinusitis are well known. Typical symptoms include nasal congestion, rhinorrhea, facial pressure, sore throat, and, in some cases, sinus tenderness (1). These infections often lead to secondary acute rhinosinusitis (ARS) due to the inflammatory response in the nasal passages and sinuses.

As regards the coronavirus infection, however, the constant and rapid mutation of this virus requires constantly learning new aspects. Initial COVID-19 cases, predominantly involving earlier SARS-CoV-2 strains, frequently presented with anosmia (loss of smell) and ageusia (loss of taste) as distinctive symptoms (16, 17). Yet, with successive mutations of the virus, changes in the spectrum of symptoms have been observed (18). The study by Whitakera et al., published in *Nature* in 2022, highlighted a decrease in anosmia and an increase in flu-like and cold symptoms, including nasal congestion and rhinorrhea (19, 20). This aligns with our findings that nasal congestion was less prevalent in COVID-19 patients compared to those with common cold or influenza.

It is believed that the SARS-CoV-2 virus more frequently manifests with dysfunction of the respiratory epithelium than with other sinonasal symptoms (21). Khan et al. found that the virus most commonly infects through the ciliated cells of the respiratory epithelium, which shed their ciliary axonemes, impairing mucociliary clearance and leading to disease progression (22). Moreover, post-viral olfactory dysfunction occurs not only in COVID-19 patients but also in 18–42% of individuals after viral URTI (23). It is thought to begin as inflammation of the nasal mucosa, causing conductive obstruction and reduced delivery of odorants to the olfactory epithelium. This condition may persist when there is direct damage to the olfactory epithelium (OE) and the olfactory bulb (24). The SARS-CoV-2 virus utilizes angiotensin-converting enzyme 2 (ACE2) for cell entry and the serine protease TMPRSS2 for S protein priming. Expression of ACE2 facilitates virus replication in the airway epithelium and increases susceptibility to infection, while TMPRSS2 contributes to the virus entry into cells and promotes its transmission (25, 26).

Differences in COVID-19 clinical outcomes may be influenced by the level of expression of the SARS-CoV-2 entry receptor genes, ACE2 and TMPRSS2, in the airways. Given the high expression and wide distribution of TMPRSS2 in human organs, ACE2 may be a key factor for limiting SARS-CoV-2 infection (27). Hence, factors that lead to an increased expression of ACE2 in host cells are likely to increase the risk of SARS-CoV-2 infection (28).

Our study appears to confirm that the most common symptom of URTI – nasal congestion – was less frequent in patients with confirmed COVID-19. This symptom was more often found in patients with common cold, influenza, and naturally in acute post-viral rhinosinusitis. Our data are consistent with findings provided in reports from many countries (e.g., Canada, France, India or the United Kingdom) (29–32). Patients who had confirmed COVID-19 infection complained less frequently of nasal discharge than those with infections of other aetiologies (Figure 1; Table 1). Moreover, the incidence was slightly lower than in studies by other researchers concerning COVID-19. Iacobucci reported that five most common symptoms in patients infected with the Omicron variant were nasal discharge, headache, fatigue, sneezing and sore throat (33). Similar findings are provided by other authors (34, 35). The data in the available literature mainly concern the first Omicron variant, hence our results may differ from these reports. This is probably related to another mutation of the virus: the Omicron super-variant. According to data provided by the Polish Ministry of Health, in Poland 128,053 infections with the Sars-CoV2 virus were detected from November to March, whereas 5,107 cases of infections with this virus were confirmed in the Warmia and Mazury region (36).

Furthermore, palpation tenderness did not reveal any significant correlation with the URTI type. Rhinoscopy findings were also not significantly different as regards these factors. This may indicate that physical examination results are not specific to any of the infections studied. Interestingly, even in COVID-19 cases, where a wide range of symptoms can present, palpation tenderness did not show a distinct pattern. This lack of specificity in palpation tenderness suggests that it cannot be reliably used to differentiate between COVID-19 and other types of URTIs.

Our study demonstrated that ARS symptoms are often present in patients with common cold, influenza and COVID-19, although their presence was least common in those with COVID-19. Clinical diagnosis of ARS in earlier COVID-19 variants has been confirmed by previous studies (37). An interesting finding is a statistically significant relationship between a history of COVID-19 or SARS-CoV-2 vaccination and the occurrence of ARS. Patients who contracted COVID-19 after a previous exposure to viral antigens more often presented with ARS symptoms compared with those in whom this was not confirmed. One explanation for this phenomenon may be a stronger activation of the inflammatory response at the most common site of virus entry into the body, i.e., the nasal epithelium, in patients who already have SARS-CoV-2 antibodies. This triggers an inflammatory cascade leading to mucosal swelling and secretion production. There is also another conclusion that may be indicated by these results: symptomatic differences between COVID-19 and other common viral URTIs become less significant with acquired immunity. These observations require further studies.

## Limitations

The limitations of our study embrace several aspects. First, although the study group was carefully selected, its size was limited to 276 patients, which may affect the overall representativeness of the results. Second, the study was conducted during only one infectious diseases season, which may limit its ability to reflect the full spectrum of seasonal variation in URTI symptoms. Third, the focus on one GP practice may limit generalisability of the obtained results to other populations and settings. Moreover, diagnosis was mostly based on clinical signs and antigen tests, which may be less precise than laboratory methods. Finally, certain factors that can impact the symptoms, such as the age of patients, the presence of comorbidities or the use of medication, were not included in the study. These factors may have influenced the presentation of symptoms but were not included in the analysis. Nevertheless, despite limitations, the results of our study serve as an important contribution to the understanding of the differences in URTI symptoms and their relationship to the infection aetiology. Further studies with larger patient groups and a longer follow-up period may help to confirm and complement our findings.

## Conclusion

Summing up, our study demonstrated significant differences in the symptoms and course of URTIs depending on their type and confirmed the influence of vaccination on the disease course, which may be significant for a routine everyday clinical practice. Although nasal congestion, nasal discharge and symptoms of sinusitis are less frequently directly associated with SARS-CoV-2 than is the case with other viruses, these symptoms still occur in a significant proportion of patients. Furthermore, our study suggests that as immunity against SARS-CoV-2 is achieved, nasal symptoms will become more frequent.

## Data Availability

The original contributions presented in the study are included in the article/supplementary material, further inquiries can be directed to the corresponding authors.
